# An updated review deciphering the anticancer potential of pentacyclic triterpene lupeol and its nanoformulations

**DOI:** 10.3389/fphar.2025.1594901

**Published:** 2025-05-09

**Authors:** Lujain A. AlMousa, Pratibha Pandey, Sorabh Lakhanpal, Ashish Kumar Kyada, Malathi. H, Priya Priyadarshini Nayak, Arif Hussain, Tarique Noorul Hasan, Reham I. Alagal, Fahad Khan

**Affiliations:** ^1^ Department of Health Sciences, College of Health and Rehabilitation Sciences, Princess Nourah bint Abdulrahman University, Riyadh, Saudi Arabia; ^2^ Centre for Research Impact and Outcome, Chitkara University Institute of Engineering and Technology, Chitkara University, Rajpura, Punjab, India; ^3^ School of Pharmaceutical Sciences, Lovely Professional University, Phagwara, Punjab, India; ^4^ Department of Microbiology, Faculty of Science, Marwadi University Research Center, Marwadi University, Rajkot, Gujarat, India; ^5^ Department of Biotechnology and Genetics, School of Sciences, JAIN (Deemed to be University), Bangalore, Karnataka, India; ^6^ Department of Medical Oncology, IMS and SUM Hospital, Siksha ‘O’ Anusandhan (Deemed to be University), Bhubaneswar, Odisha, India; ^7^ School of Life Sciences, Manipal Academy of Higher Education, Dubai, United Arab Emirates; ^8^ Department of Molecular Genetics, Sh. Tahnoon Bin Mohammed Medical City (STMC), Pure Health, Al Ain, United Arab Emirates; ^9^ Center for Global Health Research, Saveetha Medical College, Saveetha Institute of Medical and Technical Sciences, Chennai, Tamil Nadu, India

**Keywords:** lupeol, cancer, signaling pathway, pharmacokinetics, triterpene, natural compound, metabolites

## Abstract

Triterpenoids from plants are essential sources of nutraceuticals, which possess numerous positive effects on human health. Lupeol (a pentacyclic dietary triterpenoid) is commonly present in edible fruits, vegetables, and medicinal plants. Numerous investigations on the pharmacological properties of lupeol have been carried out in the past 10 years, and the results have shown that the compound has enormous pharmacological properties, including antioxidant, anti-inflammatory, and anticancer properties. Research has shown that lupeol affects the functioning of numerous molecules, including the cytokines IL-2, NFκB, IL4, IL5, cFLIP, ILβ, and Bcl-2. Our review discusses recent advancements in plant lupeol and its underlying mode of action in combating human carcinoma within the timeframe spanning from 2010 to 2024. Also, we have tried to incorporate recent studies reported till date of the finalization of this review. In order to give researchers the most recent information, highlight the limitations of pertinent research at this time, and highlight both the mechanisms of action of lupeol and recent advances in its formulations that should be strengthened in future studies.

## 1 Introduction

Triterpenes exhibit immunomodulatory properties by targeting many signaling components, including PI3K/Akt/mTOR, NF-κB, STAT3, and TLRs (Gill et al., 2016; MR Patlolla and V Rao, 2012). Triterpenes isolated from fungi and plants mainly target apoptotic processes, with additional cancer signaling pathways remaining unclear (Gill et al., 2018; Shanmugam et al., 2012). Numerous triterpenes such as 12-dioxooleana-1,9 (11)-dien-28-oic acid (CDDO), Ginsenoside, Escin, Glycyrrhetinic acid, and Oleanolic acid have been shown to produce positive effects, while some have already passed clinical trials (Gill et al., 2016; Lacaille-Dubois and Wagner, 2017). Natural triterpenoids are gaining popularity because of their diverse biological effects like cholesterol in animal cell membrane, stabilization of phospholipid bilayers by free triterpenes in plant cell membranes, making them crucial structural elements of plant membranes (Prades et al., 2011). A dietary triterpene called lupeol is one such substance that has drawn the interest of researchers, and pharmaceutical marketers worldwide (Siddique and Saleem, 2011). This article offers a thorough description of experimental research done to ascertain Lupeol’s potential as a therapeutic and chemopreventive drug for the management of cancer and inflammation.

Lupeol has been widely found in fruits (figs, guava, strawberries, red grapes, date palm and mulberries) and reported in numerous medicinal plants including *Shea butter, Celastrus paniculatus* Willd.*, licorice, Tamarindus indica* L.*, Leptadenia hastata* Vatke*, Crataeva nurvala* Buch. Ham.*, Zanthoxylum riedelianum* Engl.*, Allanblackia monticola* Mildbr. ex Staner, *Himatanthus sucuuba* (Spruce ex Müll.Arg.) Woodson*, Emblica officinalis* Gaertn.*, Bombax ceiba* L.*, Sebastiania adenophora* Pax and K. Hoffm. and *Aegle marmelos* (L.) Corrêa Several experimental researches have proven the potential efficacy of lupeol in chemoprevention (Sen et al., 2024). Numerous elements linked to cancer are effectively targeted by lupeol, which affects the processes of apoptosis, progression, and proliferation. Controlling signaling pathways such as PI3K/AKT/mTOR, Wnt, RAS/BRAF/MEK, and ERK mediate these effects (Tsai et al., 2016; Chaturvedi et al., 2008; Saleem et al., 2005; Prasad et al., 2009; Li et al., 2022). Notably, lupeol has selective anticancer effects, mainly targeting cancer cells and sparing healthy ones. This selectivity is significant since it reduces the possibility of harmful effects on healthy cells.

Experimental research has shown that lupeol possesses significant anticancer potential, encouraging outcomes for a broader range of human carcinomas such as melanomas, colorectal, and prostate carcinoma (Saleem et al., 2005; Prasad et al., 2009; Li et al., 2022; Saleem et al., 2008; Parvez et al., 2025a). Lupeol inhibits cell proliferation, reduces angiogenesis, and induces apoptosis as part of its anticancer effects. It has been found to interfere with the PI3K/Akt and Wnt/β-catenin signaling pathways, that are essential for survival and metastasis of cancer cells and key apoptosis proteins (Bax and Bcl-2) (Sen et al., 2024; Chaturvedi et al., 2008). Despite numerous reviews analyzing the therapeutic potential of lupeol, none have consolidated recent discoveries about its anticancer efficacy thus to give researchers the most recent information highlighting both the mechanisms of anticancer action of lupeol and recent advances in its formulations. Furthermore, there is currently insufficient clinical evidence to support its usage in cancer patients, which requires attention. We have included all experimental findings related to the anticancer properties of lupeol, including the application of nanotechnology to augment its efficacy and its ability to mitigate chemotherapy-induced drug resistance in cancer patients. In order that should be strengthened in future studies. Table 1 summarizes some of the plants as a major source of lupeol triterpene. Methodology used in this review includes literature on anticancerous efficacies of lupeol using PubMed and Google Scholar to collect studies having following terms including “lupeol” and “cancer,” We have considered the timeframe spanning from 2010 to 2024 and included all *in vitro, in silico, in ovo,* and *in vivo* data included in this review.

**TABLE 1:** Plants and their different parts as a potential source of lupeol.

Plant scientific name	Family	Part of the plant	References
*Arctium lappa* L.	Asteraceae	Leaf and whole plant	Taleb Agha et al. (2020)
*Arundo donax* L.	Poaceae	Leaf	Shakeri and Ahmadian (2014)
*Albizia lebbeck* (L.) Benth.	Fabaceae	Flower	Abd El-Ghany et al. (2015)
*Arbutus unedo* L.	Ericaceae	Leaf	Miguel et al. (2014)
*Alnus glutinosa* (L.) Gaertn.	Betulaceae	Bark	Felföldi-Gáva et al. (2012)
*Arctostaphylos uva-ursi *(L.) Spreng.	Ericaceae	Leaf and whole plant	Sheeri (2011)
*Aloe vera* (L.) Burm.f.	Asphodelaceae	Leaf and whole plant	Almeida et al. (2023)
*Aspidosperma quebracho-blanco *Schltdl.	Apocynaceae	Bark	Zunino et al. (2020)
*Apocynum cannabinum* L.	Apocynaceae	Root	Patil et al. (2018a)
*Bidens pilosa* L.	Asteraceae	Whole plant	Lima Silva et al. (2011)
*Cajanus cajan* (L.) Huth	Fabaceae	Root	Olubodun-Obadun et al. (2023)
*Camellia sinensis* (L.) Kuntze	Theaceae	Oil and Seed	Zhou et al. (2019)
*Capsicum annuum* L.	Solanaceae	Seed	Silva et al. (2018)
*Calendula officinalis* L.	Asteraceae	Flower	Niżyński et al. (2015)
*Camellia japonica* L.	Theaceae	Seed	Majumder et al. (2020)
*Ceiba pentandra* (L.) Gaertn.	Malvaceae	Bark	Das et al. (2021)
*Cucurbita pepo* L.	Cucurbitaceae	Flower	Abdelmonsef et al. (2024)
*Caesalpinia pulcherrima* (L.) Sw.	Fabaceae	Root	Vuyyala et al. (2021)
*Crataegus rhipidophylla* Gand.	Rosaceae	Bark	Belorgey et al. (2023)
*Crataegus laevigata* (Poir.) DC	Rosaceae	Bark	Edorh Tossa et al. (2023)
*Cucumis sativus* L.	Cucurbitaceae	Seed	Da Silveira et al. (2010)
*Coccinia grandis* (L.) Voigt	Cucurbitaceae	Fruit	Hasan and Sikdar (2016)
*Cucumis melo* L.	Cucurbitaceae	Seed	Fahamiya et al. (2016)
*Duchesnea indica* (Andrews) Teschem.	Rosaceae	Whole plant	Gupta and Prakash (2020)
*Daucus carota* L.	Apiaceae	Root	Mamdouh et al. (2024)
*Elaeagnus pungens* Thunb.	Elaeagnaceae	Leaf	Nazir et al. (2020)
*Gentiana lutea* L.	Gentianaceae	Root	Tsukurova et al. (2017)
*Dipteryx odorata* (Aubl.) Forsyth f.	Fabaceae	Bark	Sousa et al. (2022)
*Eupatorium odoratum* Walter	Asteraceae	Leaf	Liu et al. (2015)
*Glycyrrhiza glabra* L.	Fabaceae	Whole plant	Hayashi et al. (1988)
*Ficus carica* L.	Moraceae	Leaf	Ivanov et al. (2018)
*Glycine* max (L.) Merr.	Fabaceae	Seed	Davidyants (2024)
*Gaultheria procumbens* L.	Ericaceae	Shoot	Liu et al. (2013a)
*Holarrhena pubescens* Wall. ex G.Don	Apocynaceae	Bark	Zahara et al. (2020)
*Helianthus annuus* L.	Asteraceae	Shoot	Galisteo et al. (2024)
*Juniperus communis* L.	Cupressaceae	Stem	Singh et al. (2024)
*Hemidesmus indicus* (L.) R.Br.	Apocynaceae	Root	Dixit et al. (2022)
*Maclura pomifera* (Raf.) C.K.Schneid.	Moraceae	Bark, fruit, Root and whole plant	Filip et al. (2015)
*Lycopersicon esculentum* Mill.	Solanaceae	Whole plant	Zia et al. (2023)
*Lawsonia inermis* L.	Lythraceae	Whole plant	Singh et al. (2015)
*Morus alba* L.	Moraceae	Whole plant	Böszörményi et al. (2009)
*Ligustrum japonicum* Thunb.	Oleaceae	Seed	Bouknana et al. (2018)
*Menyanthes trifoliata* L.	Menyanthaceae	Root	Latté (2020)
*Myrica cerifera* L.	Myricaceae	Leaf and Root	Ahmad et al. (2022)
*Phoenix dactylifera* L.	*Aracaceae*	Stem	AbouZeid et al. (2024)
*Oenothera biennis* L.	Onagraceae	Fruit	Munir et al. (2017)
*Sweetia panamensis* Benth.	Fabaceae	Whole plant	Maldini et al. (2009)
*Panax quinquefolius* L.	Araliaceae	Seed oil	MA Domingues et al. (2014)
*Skimmia arborescens *T.Anderson ex Gamble	Rutaceae	Leaf	Epifano et al. (2015)
*Stevia rebaudiana* (Bertoni) Bertoni	Asteraceae	Leaf	IZZATI et al. (2019)
*Swertia chirata* Buch.-Ham. ex Wall.	Gentianaceae	Whole plant	Kaur et al. (2019)
*Panax ginseng* C.A.Mey.	Araliaceae	Seed oil	Lee et al. (2020)
*Vitis vinifera* L.	Vitaceae	Root, leaf and stem	Burdziej et al. (2019)
*Spartium junceum* L.	Fabaceae	Flower	Stark (1951)
*Tephrosia purpurea* (L.) Pers.	Fabaceae	Whole plant	Khatoon et al. (2019)
*Vitellaria paradoxa* C.F.Gaertn.	Sapotaceae	Seed oil and whole plant	Sirignano et al. (2021)
*Thevetia peruviana* (Pers.) K.Schum.	Apocynaceae	Whole plant	Gupta et al. (2011)
*Verbascum thapsus* L.	Scrophulariaceae	Root, Leaf and stem	Das et al. (2024)
*Vernonia cinerea* (L.) Less.	Asteraceae	Seed	Asha et al. (2016)
*Trilisa odoratissima* (J.F.Gmel.) Cass.	Asteraceae	Leaf	Banu (2024)
*Verbena officinalis* L.	Verbenaceae	Whole plant	Ahmed et al. (2017)
*Ulex europaeus* L.	Fabaceae	Whole plant	Carvajal et al. (2021)

Detailed biosynthesis of lupeol can be extracted from the study reported by Sohag et al. (2022). In brief, Lupeol (C_30_H_50_O; 426.7174 (g/mol) biosynthesis reported in cytosol *via* stepwise formation of mevalonate (MVA), diphospho mevalonate, isopentenyl pyrophosphate (IPP), and dimethylallyl pyrophosphate (DMAPP) and farnesyl pyrophosphate (FPP) from acetyl CoA. These chains of reactions are catalyzed by enzyme namely farnesyl pyrophosphate synthase (FPS). Next, squalene synthase (SQS) converts FPP into squalene which then gets oxidized to 2, 3-oxidosqualene *via* squaleneepoxidase (SQE). Later oxidosqualene gets cyclized by lupeol synthases (LUS) enzyme to form the lupenylcation which finally gets converted into lupeol through deprotonation of the29-methyl group (Phillips et al., 2006).

## 2 Pharmacokinetics and bioavailability of lupeol

The activity intensity and biological potential of a medicinal drug has been closely correlated to its absorption, excretion, distribution, and metabolism phenomenon in the human body. Therapeutic efficacy of lupeol is limited due to issues related with its bioavailability and pharmacokinetics that helps in determining its clinical relevance. With its limited water solubility, lupeol does not get easily dissolve in gastrointestinal fluids, which restricts its uptake and impedes oral absorption *via* intestinal epithelium. Lupeol’s lipophilicity allows it to diffuse passively, but overallabsorption and bioavailability are still limited (Gallo and Sarachine, 2009; Banu, 2024). Following oral administration of lupeol (200 mg/kg) to female CD-1 strain mice, a mono-compartmental model was used by MassLinx (Waters Corp.) software to calculate several pharmacokinetic parameters. Additionally, their findings showed that lupeol was removed mainly by feces, with a maximum elimination time (12 h) and value of 163.28 (Cháirez‐Ramírez et al., 2019). Furthermore, the pharmacokinetics of lupeol was investigated in several varied investigations to determine drug interactions, pharmacokinetic characteristics, and protein binding. Both the mouse model (Cháirez‐Ramírez et al., 2019) and the rats (Priyanka et al., 2017) were used to investigate pharmacokinetic parameters. Additionally, their findings showed that the animals rapidly and well-absorbed lupeol (class II BCS compound). To address these constraints, other competent drug delivery processes and chemical changes were explored to improve lupeol solubility and absorption. Priyanka et al. examined the impact of solid lipid nanoparticles (SLN) on inadequate lupeol bioavailability reported in *Ficus religiosa L.* extract. Following oral administration of this extract suspension corresponding to lupeol (50 mg/kg), values of Cmax (178.61 ± 24.6 ng/mL), Tmax (6 h), AUC0−24 (1,068.46ng × h/mL) and T1/2 (7.3 h) were recorded. Conversely, following the oral administration of SLN loaded *F. religiosa L.* extract; all parameters exhibited enhanced parameters (Priyanka et al., 2017). Khata and More have developed a method utilizing LC-MS/MS to measure lupeol in rat plasma. The mean pharmacokinetic characteristics of lupeol in wistar rat plasma following oral (30 mg/kg) and intravenous administration (1 mg/kg doses) were determined, indicating lower bioavailability *via* the oral route (below 1%) (Khatal and More, 2019).

The solubility and bioavailability of lupeol have been demonstrated to be improved by lipid-based formulations, *viz.* SLNs and nanoemulsions. The stability and targeted administration of lupeol could be improved by investigating additional cutting-edge drug delivery methods, such as micelles, liposomes, and polymeric nanoparticles (Kaps et al., 2021; Gupta et al., 2020; Jameel et al., 2024; Parvez et al., 2025b). Chemical alterations to lupeol molecule, including hydrophilic group incorporation and biocompatible polymer conjugation enhance its pharmacokinetic efficacy and solubility. These alterations augment its efficacy for clinical use. Long-term usage of lupeol may raise safety concerns and therapeutic benefits due to its high lipophilicity, which allows it to mount up in lipid-rich organs (liver, adipose tissue, and possibly CNS) (Ruiz-Rodríguez et al., 2017; Malinowska et al., 2015; Zhang et al., 2019). Lupeol’s strong affinity for plasma proteins, mainly albumin, may restrict its therapeutic efficacy but act as a slow-release reservoir (Rios et al., 2012). High lipophilicity of lupeol permits it to accumulate in lipid-rich organs such as adipose tissue, liver, and central nervous system, providing both limitations associated with safety and therapeutic benefits associated with its prolonged usage. Lupeol undergoes significant hepatic metabolism, involving Phase I (oxidation *via* CYP450 enzyme) and Phase II (conjugation with glutathione, sulfate or glucuronic acid), resulting in increased hydrophilic metabolites formation for efficient excretion. The biological activity of these metabolites is variable; some may preserve therapeutic properties, whereas others may exhibit reduced or no action, thereby diminishing the overall therapeutic efficiency of lupeol (Banu, 2024; González-Ponce et al., 2018; Seifu et al., 2012; Qin et al., 2024).

Lupeol and its metabolites are primarily excreted by the biliary system, with a portion also removed *via* kidneys. Enterohepatic recirculation, wherein reabsorption of conjugated metabolites occurs and get excreted into bile, that may extend half-life of lupeol; yet, the significance of this phenomenon in humans remains uncertain (Luo et al., 2022; Gallo and Sarachine, 2009). Numerous studies have displayed low oral bioavailability of lupeol due to its low solubility and fast metabolism, thereby restricting its therapeutic usage during oral administration (Patil et al., 2018b). Nanoparticle formulations have enhanced the bioavailability, solubility, and stability of lupeol, leading to improved treatment outcome and increased plasma concentrations. Lipid-based delivery systems, including liposomes and SLN, have enhanced lupeol bioavailability *via* increasing absorption and reducing metabolism (first-pass) (Sharma et al., 2020; Kumar et al., 2022). Co-administration of bioavailability enhancers (such as piperine) inhibited metabolic enzymes and enhanced intestinal absorption, has been further investigated to augment lupeol bioavailability (Stielow et al., 2023). Other administration routes, including intravenous (or intramuscular injections) and transdermal patches, have been explored to bypass the gastrointestinal tract and metabolism, significantly enhancing bioavailability (Hedaya, 2024; Morales et al., 2017; Fernald and Kurokawa, 2013). To optimize lupeol’s therapeutic efficacy, pursuing research to improve its pharmacokinetic profile using innovative formulation technologies, especially those incorporating nanotechnology, is essential. Moreover, whereas preclinical studies yield substantial insights into the pharmacokinetics of lupeol, more clinical trials are necessary to corroborate these results in humans. To appropriately endorse its therapeutic application, these investigations should examine lupeol’s metabolism, absorption, excretion, and distribution across diverse populations. Future pharmacokinetic investigations on lupeol are essential to enhance its viability in clinical applications. Since the primary goal of this study is to highlight the developments in the use of lupeol as an anticancer agent, other sections will discuss the effectiveness of lupeol specifically for cancer.

## 3 Anticancer potential of lupeol

**FIGURE 1:**
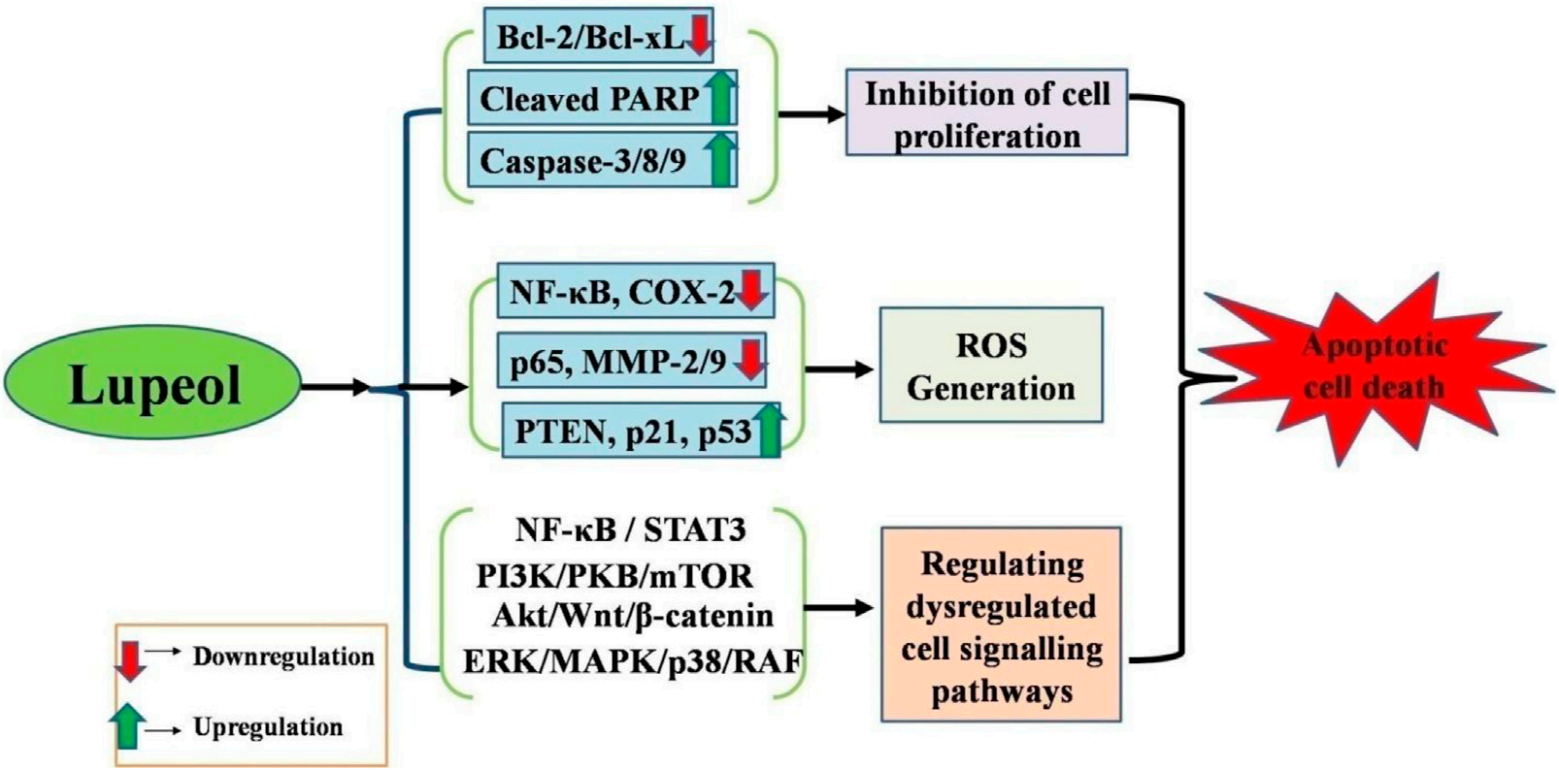
Anticancer potential of lupeol against different human carcinomas *via* (i) Apoptosis induction, (ii) Growth inhibition, (iii) Increased ROS generation, (iv) Regulation of dysregulated cell signaling pathways.

Apoptosis (programmed cell death) is a conserved mechanism essential for tissue growth and equilibrium in organisms. In pathological conditions like cancer, cells forfeit their ability to undergo apoptosis, leading to unregulated growth. Cancer cells often exhibit overexpression of several proteins essential for inhibiting the activation of the apoptotic cascade. Cells can circumvent programmed cell death through many mechanisms, one of which involves the overexpression of anti-apoptotic molecules (Singh and Lim, 2022; Chaudhry et al., 2022). Lupeol has garnered significant attention for its anticancer effects (Figure 1). Liu and colleagues (2021) have concluded that lupeol possesses considerable promise for preventing and treating many cancers impacting the bone, liver, lung, colon, rectum, and bladder. Lupeol exhibits anticancer properties by regulating apoptosis, blocking the invasion of cancerous cells, decreasing cell proliferation, and potentially increasing the sensitivity of cancerous cells to chemotherapy and radiotherapy (Liu et al., 2021).

Numerous studies have displayed apoptotic induction by lupeol in several cancer cells *via* targeting crucial proto-oncogenes and apoptotic genes. Cancer cells frequently circumvent apoptotic mechanisms owing to a disparity between pro-apoptotic and anti-apoptotic proteins. Lupeol therapy may elevate reactive oxygen species levels, augment the Bax/Bcl-2 ratio, and facilitate PARP (poly (ADP-ribose) polymerase) cleavage. This mechanism can activate caspases and commence the apoptotic execution phase (Dalimunthe et al., 2024; Park et al., 2024; Cho et al., 2024; Aly et al., 2024). Multiple studies have illustrated considerable efficacies of lupeol in the treatment of various carcinomas; therefore, we have delineated the molecular pathways underlying the anticancer efficacy of lupeol in diverse human carcinomas.

### 3.1 Breast cancer

Triple-negative breast cancer (TNBC) represents the most aggressive variant of breast cancer, characterized by restricted treatment alternatives and dismal prognosis. The anti-tumor efficacy of lupeol against breast cancer cells and the associated processes were investigated by various *in vitro* and *in vivo* experiments (Table 2). These research findings suggested that triterpenoids may impede TNBC through many signaling mechanisms. Furthermore, experimental evidence demonstrated that triterpenoids displayed remarkable anti-proliferative and anti-metastatic properties and the capacity to induce autophagy by blocking various signaling pathways, including the Akt-mTOR pathway (Saini et al., 2019). Lupeol isolated from ethanolic extract of *Bombax ceiba* (stem bark) *via* normal phase column chromatography. Out of which fourteen derivatives were synthesized and assayed for their antitumor potential against A549, MDA MB-231, and HeLa cells. Amongst all the synthesized derivatives of lupeol, pyrimidine-2(5H)-thione derivative (15) displayed significant growth inhibition in a dose dependent manner in all the selected cancer cells [MDA MB-231 (IC_50_ 27.13 µM), A549 (IC_50_ = 46.27 µM), and HeLa (IC_50_ = 45.95 µM)] (Wu et al., 2023).

**TABLE 2:** Lupeol efficacy against breast cancer cells either in alone or in combination with other drugs.

Breast cancer	Mode of action (dose dependent manner)	References
MDA MB-231 (IC_50_ 27.13 μM)	Growth inhibition	Saini et al. (2019)
MCF-7 (IC_50_ = 80 μM)	Altered cellular morphology Reduced cancer cell population Downregulated Bcl-2/Bcl-xL protein expressions	Pitchai et al. (2014)
MCF-7 (IC_50_ = 42.55 μM) MDA-MB-231 (IC_50_ = 62.24 μM) HFF (IC_50_ = 65.9 μM)	Synergistically increased the antiproliferative effect of doxorubicin Reduced cell migration induced by doxorubicin Elevated apoptosis induction Increased caspase-3 activation Reduced MMP-9 expression levels	Malekinejad et al. (2022)
MDA-MB-231 (IC_50_ = 45.67 μM) MDA-MB-231 tumor-bearing mice	Anti-metastatic *via* inhibiting the AKT-mTOR pathway Inhibited colony formation Activating autophagy Suppressed epithelial-mesenchymal transition *via* autophagy induction Inhibited tumor weight and growth	Zhang et al. (2022)
TNBC cell line MDA-MB-231 and murine TNBC cell line 4T1 (Lupeol+5FU) Lupeol (IC_50_ = 8.651 μM) 5FU (IC_50_ = 10 μM	Mount c-MET and EphA2 activation Significant anticancer effects *in vitro,* *in vivo*, and in *ex vivo* Overcome c-MET/EphA2 mediated chemoresistance Apoptotic induction *via* reduced anti-apoptotic (Bcl2) protein EMT reversal Reduced phosphorylation of EphA2, c-MET, and their downstream molecules Reduced Ki-67 and increased caspase-3	Mitra et al. (2023)
β-Carotene and lupeol (IC_50_ = 54.8 μM) β-Carotene IC_50_ = 101.5 μM) Lupeol (IC_50_ = 84.08 μM) EAC tumor-bearing mice	Most potent inhibition Exhibited moderate inhibition β-carotene and lupeol reduced viable cell count and tumor’s volume and weight and increased lifespan Significant enhancement in lipid peroxide levels in liver tissue Increased SOD levels	Jana et al. (2023)
MDA-MB-231 cells	Downregulated expression levels of NF-κB, COX-2, p65, MMP-2/9. NF-κB signaling pathway	Wang et al. (2016)

Although chemotherapy is still the primary treatment for cancers, issues like poor chemotherapy outcomes and drug resistance susceptibility are present. Natural compounds have various pharmacological potential that are significant sources for drug discovery in tumor treatment. Integrating chemotherapeutic agents with natural substances is becoming recognized as an important technique and developmental trajectory for cancer treatment (Castañeda et al., 2022; Fatma et al., 2024). Fatma et al. (2024) identified 20 trials and proved that Lupeol affects tumor volume and weight. Moreover, Lupeol and other chemotherapeutic drugs (such as cisplatin, oxiplatin, 5-fluorouracil and sorafenib) had superior results compared to their administration in alone or in combination. Lupeol further interacted with multiple signaling molecules and pathways to have an anti-cancer action (Fatma et al., 2024). Another study evaluated the anticancer potential of lupeol, isolated from *Elephantopus scaber* L. (leaves), and reported significant inhibition of MCF-7 cell viability (IC_50_ = 80 μM), cell death induction, altered cellular morphology and reduced cancer cell population with no effects on normal cells. Lupeol treatment induced apoptosis *via* downregulating Bcl-2/Bcl-xL protein expressions in MCF-7 cells in a dose dependent manner (Pitchai et al., 2014). These findings indicated lupeol as a potent anticancer adjuvant in breast cancer management. The anticancer properties of lupeol are attributed to its antiproliferative, antimigrative, and apoptotic effects. Lupeol synergistically increased the antiproliferative effect of doxorubicin on three different cells, including MCF-7 (IC_50_ = 42.55 μM), MDA-MB-231 (IC_50_ = 62.24 μM), and HFF (IC_50_ = 65.9 μM) cells in a dose dependent manner. Lupeol treatment resulted in reduced cell migration induced by doxorubicin. Apoptotic cells were elevated in lupeol and doxorubicin-treated cells with increased caspase-3 activation. This combinatorial therapy reduces MMP-9 expression levels in both MCF-7 and MDA-MB-231 cells in a dose dependent manner (Malekinejad et al., 2022).

A separate *in silico* investigation suggested that lupeol may impede TNBC by targeting various cellular signaling pathways. Furthermore, experimental results (both *in vitro* and *in vivo*) confirmed that lupeol displayed considerable anti-metastatic and autophagy-inducing properties by inhibiting the AKT-mTOR pathway and activating autophagy, suppressing epithelial-mesenchymal transition. This study confirmed the autophagy-inducing capability of lupeol in TNBC cells, suggesting its usage as a dietary supplement or prospective therapeutic alternative for TNBC (Zhang et al., 2022). In patients receiving 5FU, hyperactivation of c-MET and type-A receptor 2 (EphA2) was associated with a decreased risk of disease-free survival. Nevertheless, the invasive and tumorigenic efficacy of TNBC cells was significantly reduced upon silencing both of these genes, suggesting that a dual-target approach is feasible. A synergistic approach utilizing 5FU and Lupeol has shown encouraging anticancer effects *in vitro, in vivo*, and in *ex vivo* (patient-derived tumor culture model). Even in the presence of HGF, this synergistic regimen is adequate whichmountc-MET and EphA2 activation. These data provided the basis for the clinical validation of this combinatorial therapeutics for TNBC patients (Mitra et al., 2023).

A supplementary investigation corroborated the utilization of synergistic methods as an innovative strategy to surmount monotherapy resistance in breast cancer treatment. The combination of β-Carotene and lupeol had the most potent inhibition (IC_50_ = 54.8 μM) compared to the standard inhibitor, whereas β-Carotene (IC_50_ = 101.5 μM) and lupeol (IC_50_ = 84.08 μM) exhibited moderate inhibition in a dose dependent manner. The treated animal groups’ hematological, biochemical, and tissue antioxidant markers returned to normal levels. β-carotene and lupeol reduced viable cell count and tumor’s volume and weight and extending the lifespan of EAC tumor-bearing mice (Jana et al., 2023). An extract of *Euphorbia fischeriana* Steud. was utilized to investigate the inhibitory effects of lupeol on invasion of MDA-MB-231 cells. Lupeol markedly impeded the invasion of MDA-MB-231, as demonstrated in all experimental assays, including wound healing, cell adhesion, and transwell assays. Lupeol suppressed MDA-MB-231 cells migration by downregulating the expression levels of NF-κB, COX-2, p65, MMP-2/9. This investigation confirmed the inhibitory efficacy of lupeol on the nuclear NF-κB signaling pathway in breast cancer in a dose dependent manner (Wang et al., 2016). These investigations may validate the anticancer activity of lupeol, either alone or in combination with other natural or chemotherapeutic agents, and offer compelling evidence for its use as an anti-breast cancer agent.

### 3.2 Cervical and ovarian cancer

Lupeol has been insufficiently investigated in cervical and ovarian cancer, hence offering fresh opportunities for future researchers to examine its anticancer potential in these malignancies. In HeLa cells, lupeol administration drastically decreased cell proliferation and viability by triggering S-phase cell cycle growth arrest and reducing the expression of CDKs and S-phase cyclin. Lupeol administration also elevated the expression levels of cyclin-dependent kinase inhibitors, specifically p21 (at both protein and mRNA levels). Additionally, lupeol prompted apoptosis and elevated the expression of apoptotic markers, including cleaved PARP and Bax/Bcl-2 ratio in a dose dependent manner. Additionally, increased mitochondrial superoxide production and decreased healthy mitochondrial mass were seen in HeLa cells treated with lupeol. The findings indicate that lupeol may serve as an efficacious chemotherapeutic treatment for cervical cancer, owing to its growth-inhibitory properties *via* the activation of S-phase cell cycle growth arrest and death (Prasad et al., 2018). Lupeol suppressed growth and induced apoptosis in ovarian cancer cells *via* inactivating the PI3K/PKB/mTOR signaling pathway. Lupeol potentially enhanced cell apoptosis, reduced cell viability, triggered G1 phase growth arrest and decreased the ratio of phospho-AKT to PKB and phospho-mTOR to mTOR in a dose dependent manner (Chen and Zhang, 2021).

### 3.3 Gastric cancer

Gastric cancer is histologically identified using endoscopic biopsy and staged utilizing laparoscopy, CT, PET, and endoscopic ultrasound. The disease exhibits significant molecular and phenotypic heterogeneity (Smyth et al., 2020). The primary intervention for early stomach cancer is endoscopic resection. Unresectable stomach carcinoma is managed with surgical intervention that must encompass D2 lymphadenectomy. Perioperative (or adjuvant chemotherapy) enhances survival in patients with advanced malignancies. Advanced stomach cancer is managed with successive chemotherapy regimens, commencing with a platinum and fluoropyrimidine combination in the first line (survival is under 1 year). Approved targeted therapy for gastric cancer comprises nivolumab, trastuzumab, ramucirumab, and pembrolizumab (Van Cutsem et al., 2016; Hartgrink et al., 2009). Researchers have employed lupeol as a promising lead candidate for treating gastric carcinoma and colorectal and renal cell carcinomas to address these limitations (Table 3). Lupeol showed efficacy against various gastric cancer cell lines (HGC27, BGC823, and N87) by decreasing their proliferation through natural killer cell activity enhancement. Lupeol further elevated IFN-γ, CD107a, and PFP expression levels by activating the PI3K/Akt/Wnt/β-catenin signaling pathway in a dose dependent manner (Wu et al., 2013).

**TABLE 3:** Lupeol efficacy against gastric cancer cells either in alone or in combination with other drugs.

Gastric cancer	Mode of action (dose dependent manner)	References
N87, BGC823 and HGC27 (gastric cancer) cells (Lupeol = 0.1 μg/mL to 200 μg/mL)	Upregulated NK cells expression Growth inhibition Increased expression of CD107a (lysosomal membrane glycoprotein), IFN-γ and Perforin Activated PI3K/Akt/Wnt/β-catenin signaling pathway	Wu et al. (2013)
KKU-M213 and RMCCA-1 (CCA) cells Lupeol + stigmasterol Lupeol (IC_50_ = 10) β-sitosterol (IC_50_ = 12.5) stigmasterol (IC_50_ = 21.1 μM) Cholangiocarcinoma xenograft model	Inhibited endothelial cell migration Inhibited formation of capillary network Downregulated TNF-α Inhibited VEGF signaling Reduced tumor angiogenesis, macrophage recruitment and tumor growth	Kangsamaksin et al. (2017)
SK-RC-45 (renal cell carcinoma) cells Lupeol (LC_50_ = 40 μM)	Mitochondrial hyperfission generation Apoptosis induction Promoting autophagy-mediated selective clearance of disintegrated mitochondria	Sinha et al. (2019)
HT-29 (human colorectal adenocarcinoma) cell	Angiogenesis and cell migration inhibition Specific targeting of mutated VEGF	Ambasta et al. (2015)
Experimental bladder carcinogenesis (Lupeol = 50 mg/kg body weight) + (Diindolylmethane = 5 mg/kg body weight)	Inhibited bladder tumor growth Reduced TNF- κβ (p65) and Cox-2 expression levels Activate PTEN suppressor protein	Prabhu et al. (2016)
Colorectal cancer (DLD 1 and HCT 116) cells (Lupeol (0–60 μM))	Reduced cell viability, nuclear accumulation of β-catenin and colonogenic potential Reduced expression of Wnt target genes Apoptotic induction Inhibited β-catenin phosphorylation (at S552 and S675 sites)	Tarapore et al. (2013)
LoVo colorectal cancer cell and nude mice Lupeol + Oxaliplatin (0–150 µM)	Downregulated cell proliferation and apoptosis activation *via* ABCG2 suppression and enhanced ER stress pathway Increased phosphorylation of eIF2a (P-eIF2a) and caspase-3 Reduced tumor size	Chen et al. (2018)

A separate investigation revealed the growth-inhibitory effects of lupeol and stigmasterol, which impeded cell migration and HUVECs morphogenesis. Both drugs markedly diminished the transcript levels of TNF-α, resulting in decreased phosphorylated forms of FAK, Src, PCL, and Akt. *In vivo*, administration of lupeol and stigmasterol led to diminished development of CCA tumor xenografts and impaired tumor angiogenesis. Both the phytocompounds did not exhibit any significant toxicity in mice. The phytocompounds effectively inhibited CCA tumor growth, targeted tumor endothelial cells *via* targeting their anti-inflammatory properties, making them promising candidates for treating CCA tumors (Kangsamaksin et al., 2017). Most renal cell carcinoma (RCC) patients exhibit a significant propensity for developing resistance to radiation therapy and chemoresistance. Treatment with lupeol (LC_50_ = 40 μM) on SK-RC-45 cells for 48 h generated mitochondrial hyperfission, ultimately resulting in apoptosis, while SK-RC-45 countered this effect by promoting autophagy-mediated selective clearance of disintegrated mitochondria in a dose dependent manner. Consequently, lupeol possesses the potential to serve as an effective treatment against renal cell carcinoma through the modification of mitochondrial dynamics (Sinha et al., 2019). Angiogenesis is a defining characteristic in tumor initiation, development, and growth. Luteolin has demonstrated superior efficacy in suppressing angiogenesis in the CAM assay compared to lectin and lupeol. Luteolin showed superior efficacy in preventing HT-29 cell migration. Software analysis has selected the optimal target proteins among these bioactive compounds and VEGF was identified as one of the targets of luteolin (Ambasta et al., 2015).

A separate study examined the growth inhibitory and anti-inflammatory potential of lupeol and diindolylmethane (DIM) on the growth of experimental bladder cancer. Experimental results demonstrated that combinatorial treatment inhibited bladder tumor growth (confirmed by histopathological analysis). Moreover, there was a momentous boost in PTEN expression and a notable decline in expression level of TNF-α, NF-κB (p65), and Cox-2 in bladder tissue samples, as well as in NMP 22 from urine samples. Preventive DIM and lupeol treatment are effective Cox-2 inhibitors, activating PTEN (tumor suppressor protein) in experimental bladder carcinogenesis *via* growth inhibitory and anti-inflammatory phenomenon in a dose dependent manner (Prabhu et al., 2016).

Lupeol has demonstrated differential expression of Wnt/β-catenin signaling in human CRC (DLD 1 and HCT 116) cells, leading to decreased cell viability, increased apoptotic induction, diminished colonogenic capacity, reduced β-catenin mRNA level, and lowered expression of Wnt target genes in a dose dependent manner. It obstructed cytoplasmic translocation of β-catenin to the nucleus. Significantly, all these efficacies of lupeol were confined to cells with constitutively active Wnt/β-catenin signaling, while few were noted in cells devoid of such signaling. This work also indicated that inhibiting Wnt signaling in cells with constitutively active Wnt/β-catenin diminishes lupeol efficacy, but activating Wnt signaling enhances the cells’ sensitivity to the inhibitory effects of lupeol (Tarapore et al., 2013).

A variety of first-line chemotherapeutic agents have been employed in colorectal cancer treatment. Oxaliplatin (OXA) (alkylating cytotoxic agent) is typically administered with other chemotherapeutic agents to treat advanced stage (II and III) CRC. Cancerous cells frequently develop multidrug resistance, posing significant barrier to cancer therapy. Lupeol-treated CRC cells exhibited diminished cell viability, induced apoptosis, decreased ABCG2 expression, and triggered endoplasmic reticulum stress to provoke oxaliplatin-resistant cell death in a dose dependent manner. Anti-tumor activity of lupeol on OXA-resistant cells surpassed that observed in LoVo parental cells. Moreover, lupeol injections substantially decreased tumor size in a xenograft animal model, acting as a potent sensitizer for chemoresistance and potentially serving as a novel adjuvant therapy for chemoresistant patients (Chen et al., 2018).

Lupeol enhances the sensitivity of drug-resistant cancerous cells to clinically authorized medications. Lupeol, independently or in conjunction with approved medications, suppresses inflammation in several cancerous cells by modulating the expression levels of IFN-γ, IL-6, and TNF-α. Lupeol therapy modulated PI3K/AKT/mTOR signaling pathway and expression levels of caspases, Bcl-2, FAS, BAX, and survivin. It additionally prompted cell death in therapy-resistant cells and influenced many molecules associated with cell cycle regulation, including PCNA, cyclins, p21, CDKs, and p53 across distinct carcinomas. Lupeol enhanced sensitivity of therapy-resistant cancer cells to multiple therapeutically approved medicines by altering distinct signaling pathways associated with chemoresistance. Consequently, Lupeol may serve as an adjuvant compound with clinically approved medications to enhance efficacy and mitigate toxicity. Lupeol chemosensitizes the therapy-resistant cancer cells for the treatment of various clinically approved drugs *via* modulating expression levels of IL-6, TNF-α, and IFN-γ. Lupeol, *via* altering expression levels of Survivin, BCL-2, BAX, Caspases, and FAS, and modulating cell signaling pathways induce cell deaths among therapy-resistant cells. Thus, Lupeol might be used as an adjuvant molecule along with clinically approved drugs to reduce the toxicity and increase the effectiveness (Maurya et al., 2020).

### 3.4 Hepatocellular carcinoma

Hepatocellular carcinoma (HCC) is the second foremost cause of cancer-related mortality, and its incidence is rising worldwide. Notwithstanding progress in preventive methods, screening, and innovative technology in diagnosis and treatment, incidence and mortality persist in escalating (Llovet et al., 2022). Cirrhosis remains the significant risk factor for the onset of hepatocellular carcinoma, irrespective of its cause. Accumulating research indicates that specific natural food metabolites may serve as potential sources for preventing and treating liver cancer (Mandlik and Mandlik, 2021). Dietary triterpenoids and their active derivatives have been reported to inhibit the commencement and development of liver cancer through various phenomenon, including protection against liver carcinogens, enhancement of chemotherapeutic efficacy, inhibition of tumor cell growth and metastasis, and reduction of oxidative stress and chronic inflammation (Sureda et al., 2021). A study indicated the anticancer properties of lupeol extracted from *Avicennia marina*, which thrives in the deserts of the UAE. Lupeol administration exhibited substantial growth-inhibitory effects on MCF-7 and Hep3B cells, including parental and resistant strains. Lupeol markedly reduced BCL-2 gene expression in Hep3B cells (both parental and resistant), resulting in the triggering of apoptosis and activation of executioner caspase-3. Lupeol exhibited no significant impact on monocyte proliferation; however, it increased growth arrest at G1 phase and reduced apoptotic rates of monocytes (after 48 and 72 h), suggesting a lack of immuno-inflammatory responses. Lupeol can be regarded as a promising, effective, and safe anticancer drug, notably against Hep3B cancer cells (Eldohaji et al., 2021).

Lup-20 (29)-en-3b-ol (lupeol) impeded self-renewal capacity of liver tumor-initiating cells found in both HCC cells and clinical hepatocellular carcinoma samples, as evidenced by hepatosphere formation. Moreover, lupeol suppressed tumorigenicity in nude mice and decreased CD133 expression level. Furthermore, lupeol enhanced the sensitivity of HCC cells to chemotherapeutic drugs *via* PTEN/AKT/ABCG2 pathway. PTEN is essential for self-renewal and chemoresistance of liver tumor-initiating cells; PTEN downregulation by lentiviral method negated impact of lupeol on liver tumor-initiating cells. In chemoresistant HCC tumor model (*in vivo*), lupeol significantly reduced tumor size of MHCC-LM3 HCC cell line-derived xenografts, with effects comparable to those of combined cisplatin and doxorubicin therapy. Lupeol demonstrated synergistic efficacies without negatively impacting body weight when administered alongside chemotherapy agents. These data indicated that lupeol may be an efficient dietary phytochemical targeting liver T-ICs (Lee et al., 2011). A different group documented the anticancer effectiveness of lupeol against SMMC-7721 cells. Lupeol treatment of SMMC-7721 cells demonstrated reduced proliferation and clonogenic survival when exposed to γ-radiation. It additionally prompted cellular accumulation during the G2/M phase in conjunction with γ-radiation. Consequently, lupeol-sensitized SMMC-7721 cells subjected to γ-radiation to undergo apoptosis and displayed activated apoptotic proteins (PARP and caspase-9). In HCC xenograft model, co-administration of lupeol in conjunction with radiation resulted in notable delay in tumor growth in comparison to either radiation or lupeol administered alone, which was well tolerated (Jin et al., 2015).

Constitutive STAT3 activation has been associated with angiogenesis and proliferation in numerous malignancies, including HCC. Lupeol substantially inhibited the constitutive activation of STAT3 phosphorylation (at tyrosine 705 residue) and the phosphorylation of Src and JAK 1/2. Lupeol therapy significantly elevated the mRNA and SHP-2 protein expression levels. The suppression of SHP-2 negated the inhibitory potential of lupeol on STAT3 activation. Lupeol treatment further reduced the expression levels of other STAT3-regulated genes and inhibited STAT3 binding with VEGF promoter. Furthermore, lupeol strongly inhibited growth of several HCC cells, correlating with marked increase in apoptosis (Siveen et al., 2014). In conjunction with S14161, Lupeol demonstrated a synergistic anticancer impact, leading to the chemosensitization of HCC cells to low doses of lupeol. In HCC model, lupeol and S14161 (S14161, a newly identified PI3-Kinase inhibitor *in vitro* and *in vivo*) collaboratively suppressed tumor development while activating the PI3-kinase/AKT pathway, resulting in a tumor progression without adversely affecting body weight in a dose dependent manner. Consequently, combining a PI3-kinase inhibitor with lupeol synergistically enhanced anti-tumor efficacy of lupeol and represented a viable method for hepatocellular carcinoma therapy. Inhibition of PI3K synergistically enhanced the anti-tumor efficacy of lupeol in HCC carcinoma (Liu F. et al., 2013).

A separate study examined the synergistic effects of geraniol and lupeol, revealing substantial antiproliferative and proapoptotic impacts on hepatocarcinoma cell lines (SMMC7721 and HepG2). Combined treatment of geraniol and lupeol led to decreased Bcl-2 expression levels and increased levels of Bax and caspase proteins and genes. Additionally, geraniol and lupeol treatment altered expression levels of both c-Jun NH2-terminal kinase and p38 phosphorylation, indicating their involvement in MAPK signaling, thereby suggesting their potential utility in anti-liver cancer treatment (Shen et al., 2019). A separate group investigated the anti growth potential of lupeol on HCCLM3 cells by PARP breakage and caspase-3 activation. Lupeol-induced apoptosis correlates with diminished protein expression of Ser-9-phosphorylated GSK-3β and brain-derived neurotrophic factor (BDNF) and a concurrent reduction in expression levels of c-Myc, cyclin D1, Akt1, PI3K, and β-catenin mRNAs. Inhibition of BDNF overexpression by lupeol led to diminished p-Akt and PI3K (p110α) protein levels, along with the reactivation of GSK-3β activity in HepG2 cells in a dose dependent manner. Lupeol therapy as well suppressed LiCl-induced activation of the Wnt signaling pathway and demonstrated *in vitro* anti-invasive action in Huh-7 cells. Lupeol exposure diminishes the elevated expression levels of cyclin D1, β-catenin, and c-Myc proteins induced by LiCl. The results demonstrated that lupeol can decrease HCC cell proliferation by suppressing BDNF production and the phosphorylation of GSK-3β(Ser-9), in conjunction with the inhibition of Wnt and AKT/PI3K signaling pathways (Table 4) (Zhang et al., 2015).

**TABLE 4:** Lupeol efficacy against Hepatocellular cancer cells (HCC) either in alone or in combination with other drugs.

Hepatocellular cancer	Mode of action (dose dependent manner)	References
Hep3B liver cancer cells (IC_50_ = 50 µM)	Significant inhibition of normal and resistant Hep3B cancer cells Reduced expression of BCL‐2 gene in both the cells Increased level of cleaved caspase‐3 Growth arrest at G1 phase	Eldohaji et al. (2021)
HCC cell line (MHCC-LM3) Liver tumor tissue specimens were collected from five Patients (IC50 of 280 µM)	Inhibited hepatosphere formation Reduced self-renewal ability Suppressed tumorigenicity in nude mice Reduced expression of liver tumor-initiating cells (T-IC) marker Inhibited HCC cell growth synergistically with chemotherapeutic agents Combined lupeol and chemotherapeutic drug treatment significantly suppressed tumor growth in a chemoresistant HCC nude mouse model	Lee et al. (2011)
SMMC-7721 cells HCC xenograft model	Significantly enhanced the radiosensitivity of SMMC-7721 cells *in vitro* and *in vivo* Induced accumulation of cells in G2/M phase together with γ-radiation Activated caspase-9 and PARP Used as an adjuvant for radiotherapy in HCC	Jin et al. (2015)
HepG2 and C3A (lupeol = 50 µM)	Suppressed constitutive activation of STAT3 phosphorylation Inhibited constitutive STAT3 phosphorylation in HepG2 cells Inhibited STAT3 DNA-binding activity Suppressed constitutive activation of c-Src suppresses constitutive activation of JAK1 and JAK2 Reduces nuclear pool of STAT3	Siveen et al. (2014)
HepG2 and SMMC7721 cells Lupeol and S14161 (IC_50_ values: lupeol alone: 44.9 μmol/L, lupeol + 1 μmol/L S14161: 40.1 μmol/L, lupeol + 3 μmol/L S14161: 27.9 μmol/L). Female athymic nude mice (BALB/c-nu/nu) 20 mg/kg lupeol	Exerted synergistic antitumor effect Chemo-sensitization of HCC to low doses of lupeol Using an *in vivo* HCC model, lupeol and S14161 synergistically inhibited tumor growth without any adverse effects on body weight Activated PI3-kinase/Akt pathway Synergistically augmented anti-tumor effect of lupeol	Liu et al. (2013b)
HepG2; SMMC7721	Altered the phosphorylation level of extracellular signal-regulated protein kinase, P38, and c-Jun NH2-terminal kinases Inhibited hepatocarcinoma cell growth and apoptosis Strong anti-liver cancer therapy	Shen et al. (2019)
(HCC) HCCLM3 cells	Caspase-3 dependent activation Poly ADP-Ribose Polymerase (PARP) cleavage Suppressed HCC cell proliferation *via* inhibition of BDNF secretion and phosphorylation of GSK-3β(Ser-9) Blockade of Akt/PI3K and Wnt signaling pathway	Zhang et al. (2015)

### 3.5 Other carcinomas (oral, lung, melanoma, prostate and retinoblastoma)

A multidisciplinary approach is required to treat oral cancer since every patient has a different combination of problems that affect their quality of life and chances of survival. Multiple triterpenoids, including lupeol, exhibit anticancer efficacy against various carcinomas including oral, lung, melanoma, prostate and retinoblastoma (Table 5) (MR Patlolla and V Rao, 2012). A study showed that lupeol therapy inhibited colony formation, cell proliferation, and EGFR phosphorylation in NSCLC (Non-small cell lung cancer) cells. Furthermore, lupeol prompted apoptosis by enhancing PARP breakage and chromatin condensation and inducing growth arrest in sub-G1 phase cells. *In silico* investigations demonstrated that lupeol directly interacted with tyrosine kinase domain of EGFR. Lupeol inhibited transcriptional activation and nuclear translocation of STAT3 while downregulation of STAT3 target genes expression levels. Constitutive STAT3 activation through STAT3 Y705D overexpression inhibited lupeol induced apoptosis, indicating suppression of STAT3 activation facilitated apoptosis induction. The anticancer potential of lupeol was consistently noted in EGFR-TKI-resistant H1975 cells (nonsmoking female with non-small cell lung cancer) (EGFR L858R/T790M). Results of this trial indicated that lupeol was applicable for both EGFR TKI-naive NSCLC and advanced NSCLC exhibiting acquired resistance to EGFR TKIs (Min et al., 2019). In head and neck squamous cell carcinoma lupeol has been identified to trigger apoptosis in various carcinomas *via* extrinsic mechanism. Lupeol administration enhanced p53-mediated Bax expression and triggered intrinsic apoptotic pathways, as evidenced by cleaved caspase-3. Lupeol induced G1 cell cycle arrest by up-regulating CDKN2A expression, leading to cyclinD1 inhibition in a dose dependent manner. In *ex vivo* experimentations, lupeol administration resulted in Ki67 expression inhibition, reduction in cell viability, and simultaneous caspase-3 activation. Consequently, lupeol re-sensitized primary HNSCC tumor samples that have clinically progressed during a cisplatin therapy program (Bhattacharyya et al., 2017).

**TABLE 5:** Lupeol efficacy against other carcinomas either in alone or in combination with other drugs.

Oral cancer	Mode of action (dose dependent manner)	References
Non-small cell lung cancer (H1299, A549 and H460) cells Lupeol (100 μg/m)	Inhibited growth and colony formation Apoptotic induction *via* upregulated cleaved PARP and caspase-3, and apoptosis marker proteins Blockage of EGFR activity STAT3 inhibition *via* EGFR regulation	Min et al. (2019)
Head and neck squamous cell carcinoma *In vitro* 2D cell line model HEp-2 (IC_50_ = of 53.51 μM), UPCI:SCC-131(IC_50_ = 52.44 μM) *Ex vivo* 3D tumor explant culture	Increased expression of p53, caspase-3 and cell cycle arrest (G1 phase) Modulated expression and localization of cell cycle checkpoint regulators Activated intrinsic apoptotic pathway Inhibited growth of both naive and Cisplatin Resistant cells Upregulated CDKN2A expression and cyclinD1 inhibition	Bhattacharyya et al. (2017)
Golden Syrian hamsters models (Oral administration of Lupeol at a dose of 50 mg/kg body weight)	Inhibited the formation of oral tumors Restored status of biochemical markers Free radical scavenging property of lupeol Modulating effect on phase I and II xenobiotic metabolizing enzymes excretion of carcinogenic metabolites	Palanimuthu et al. (2012)
Nasopharyngeal carcinoma 5–8 F and CNE1 cells (Lupeol: 20 μM and 40 μM)	Induction of ferroptosis and cell apoptosis Enhanced oxidative stress Restrained immune response Cleaved caspase-3 expression ROS production and malondialdehyde level Reduction of Bcl-2, TNF-α, MMP, p-IκBα, IL-1β, NF-κB p65superoxide dismutase, and IL-6 Elevated AMPKα phosphorylation Suppressed tumorigenesis of xenografts in nude mice	Zhou et al. (2022)
Human OSCC (Lupeol + Paclitaxel)	Apoptosis induction Disruption of vasculogenic mimicry associated phenotypes Downregulation of VE-Cadherin, pERK1/2, EphA2, and MMP2 Downregulated HIF-1α/EphA2/Laminin-5γ2 cascade	Saha et al. (2023)
OSCC cell lines (UPCI:SCC131 and UPCI:SCC084)	Inhibited proliferation *via* apoptosis induction Induced EGFR phosphorylation Activation of protein kinase B, I kappa B, Cox-2, and nuclear factor kappa B	Rauth et al. (2016)
DMBA induced Syrian hamster buccal pouch (Oral administration of lupeol at a dose of 50 mg/kg body weight)	Inhibited the formation oral tumors as well as reduced the expression p53 and Bcl-2, Increased expression of Bax and caspase 3 and 9	Manoharan et al. (2012)
Human MM cell lines RPMI-7951 cells (IC_50_ = 45.54) A375 (IC_50_ = 66.59)	Selective cytotoxicity against MM cells Reduced cell viability, neovascularization and confluence Cytoskeletal alterations Apoptosis induction and inhibited migration Cellular dysmorphology Angiogenesis inhibition	Bociort et al. (2021)
Human melanoma Mel 928 and Mel 1,241 cells (Lupeol (40–60 µM) Lupeol treated mice ((40 mg/Kg body weight)	Increased caspase 3/7 activation Reduced β-catenin/TCF4 transcriptional activities of Wnt signaling, IMP 1 and MITF Reduced tumor growth in Mel 1011-implanted tumors Reduced MMP, TIMPs expression in Mel 928 tumors	Tarapore et al. (2010b)
B16 2F2 melanoma cells Melanoma-bearing mouse model	Reduced tumor growth rate Induced cell cycle arrest Reduced percentage of Ki-67- and PCNA-positive areas in the tumor tissues	Nitta et al. (2013)
A427 lung cancer cells	Apoptosis induction ROS generation and MMP dysregulation Inhibition of mTOR/PI3K/AKT signalling pathway	He et al. (2018)
A549, HepG2, and MCF-7 cancer cells Lup-20 (29)-en-3β-yl-piperazinecarboxylate-(2, 4-fluorophenyl) urea (IC50 = 3.22 μM)	Reduced cell viability Apoptosis induction Efficient anticancer efficacy	Xu et al. (2024)
A549 lung cancer cells	Targeted ERK and MEK proteins Impeded cell migration Reduced pErk1/2 and epithelial-mesenchymal transition gene expression	Bhatt et al. (2021)
LNCaP and 22Rv1 prostate cancer cells Lupeol (10–50 μM) Enzalutamide (5–20 μM)	Minimize the adverse effects and augment the efficacy of Enzalutamide Facilitated mitigating oxidative and DNA damage from enzalutamide	Khan et al. (2023)
Human retinoblastoma (WERI-Rb-1 and Y-79) cells	Reduced cell viability and triggered apoptosis Elevated Bax levels and reduced Bcl-2, Ki67, and survivin levels Inhibited spheroid formation and stem-like characteristics of retinoblastoma cells	Che et al. (2022)

Lupeol (50 mg/kg bw) administered in the buccal pouch of golden syrian hamsters completely prevented oral tumor growth and restored biochemical marker levels after DMBA-induced oral carcinogenesis. Chemopreventive potential of lupeol primarily arises from its antioxidant properties and modulatory efficacies on phase I and II xenobiotic metabolizing enzymes, facilitating excretion of carcinogenic compounds during DMBA-induced carcinogenesis in hamster buccal pouches (Palanimuthu et al., 2012). In nasopharyngeal cancer, Lupeol induced apoptosis, oxidative stress, and ferroptosis while mitigating inflammation through the AMPK/NF-κB pathway and increasing the expression levels of ROS, Bax, MDA and caspase-3. It diminished MMP, Bcl-2, TNF-α, IL-6, IL-1β, and superoxide dismutase expression levels. Furthermore, lupeol facilitated iron secretion and lipid peroxidation, results that were mitigated by the ferroptosis inhibitor (Fer-1). Lupeol markedly increased AMPKα phosphorylation and decreased the levels of p-IκBα and nuclear NF-κB p65. Furthermore, lupeol inhibited carcinogenesis in xenografts inside naked mice (Zhou et al., 2022).

Vasculogenic mimicry (VM), characterized as an endothelial cell-independent alternate pathway for nutrition and blood delivery by dysregulated tumor cells, is linked to unfavorable prognosis in OSCC (Oral Squamous Cell Carcinoma) (Salem and Salo, 2021; Hujanen et al., 2021). In human oral cancer cells, lupeol combined with paclitaxel induced apoptosis and disrupted vasculogenic mimicry-associated behaviors. The efficacy of this innovative interventional method was additionally confirmed in oral cancer patient (derived tumor explant culture model). Tumor model replicated anti vasculogenic mimicry efficacy of combined lupeol and paclitaxel *via* down-regulating HIF-1α/EphA2/Laminin-5γ2 cascade. These results revealed the molecular basis of hypoxia-induced Laminin-5γ2-driven vascular mimicry creation, underscoring that the lupeol-paclitaxel combination may represent a potential therapeutic approaches for vascular mimicry disruption in human OSCC. In human oral carcinoma, combined treatment of lupeol and paclitaxel impeded hypoxia-induced VM *via* inhibiting HIF-1α-EphA2-Laminin-5γ2 network (Saha et al., 2023). A separate study demonstrated that lupeol decreased the growth of OSCC cells by triggering apoptosis after 48 h of treatment. Concomitant activation of downstream enzymes (PKB, IκB, AKT, NF-κB), ligand-induced EGFR phosphorylation was partially inhibited. Notably, Lupeol inhibited both mRNA and protein expression levels of COX-2. Lupeol treated primary explants (derived from OSCC tissues) demonstrated a marked reduction in Ki67 proliferation relative to untreated control at 48 h. Thus, lupeol served as a potent inhibitor of EGFR signaling in OSCC, suggesting its potential role in eliciting antitumor effectiveness (Rauth et al., 2016). The evasion of apoptosis is a significant hallmark of rapidly growing tumor cells. The topical administration of 0.5% DMBA thrice weekly (for 14 weeks) in buccal pouches of golden syrian hamsters led to OSCC development. This study observed 100% tumor formation and abnormalities in apoptotic marker expression in hamsters treated alone with DMBA (is a potent carcinogen which is widely employed for studying oral tumorigenesis, leading to the development of oral squamous cell carcinoma). Oral treatment of lupeol (50 mg/kg body weight) entirely inhibited tumor (oral) development, reduced expression of p53 and Bcl-2, and elevated expression levels of Bax and caspases (3 and 9). These investigations demonstrated that lupeol suppressed DMBA-induced oral tumorigenesis through its pro-apoptotic properties in golden syrian hamsters (Manoharan et al., 2012).

Clinical and histological data indicates that melanoma evolves through a series of stages, advancing from benign proliferative lesions to primary melanomas without metastatic evidence, invasive central lesions, and finally to metastases (Herlyn, 1990). Lupeol treated melanoma cells including Mel 928 and Mel 1,241 cells (but not Mel 1,011 cells) led to diminished cell viability, induction of apoptosis, decreased clonogenic capacity, reduced β-catenin transcriptional activity, and lower expression levels of Wnt target genes. It further inhibited cytoplasmic translocation of β-catenin to the nucleus and inhibited Mel 928 tumors formation, but not those produced from Mel 1011, when implanted in athymic nude mice. Reduction in tumor development resulting from Mel 928 correlated with reduced expression levels of osteopontin, c-myc, Ki-67, and cyclin D1 in a dose dependent manner. Consequently, either independently or as an adjunct to existing medicines, lupeol may be formulated as a treatment for human melanomas exhibiting constitutive Wnt/β-catenin signaling (Tarapore et al., 2010a). Malignant melanoma, lethal skin cancer reported globally, with a limited and ineffective range of chemotherapeutic options in the advanced stages of the illness. Lupeol was investigated for its effects on two human malignant melanoma cell lines (A375 and RPMI-7951) demonstrating concentration-dependent and selective cytotoxicity, with estimated IC_50_ values of 66.59 µM for A375 and 45.54 µM for RPMI-7951. Additionally, it inhibited cell migration, decreased cell confluence, reorganized cytoskeletal components, and induced apoptosis-specific nuclear characteristics in a dose dependent manner. *In ovo (*treatments or the processes used on developing embryos inside the egg), lupeol inhibited angiogenesis by diminishing neovascularization development (Bociort et al., 2021). Lupeol treated Mel 928 and Mel 1,241 cancer cells led to enhanced activation of caspase 3/7 and diminished the β-catenin/TCF4 transcriptional activity associated with Wnt signaling and its downstream targets, specifically IMP 1 and MITF. It additionally suppressed Wnt signaling activation by diminishing nuclear β-catenin levels. Lupeol therapy displayed a nonsignificant reduction in tumour development in Mel 1011-implanted tumors. In contrast, a notable reduction was seen in expression levels of Wnt targets, MMPs, and TIMPs in Mel 928 tumors derived from lupeol-treated mice, indicating its possible impact on metastatic dissemination in a dose dependent manner (Tarapore et al., 2010b).

Tumor-suppressive effects (both systemic and local injections) of lupeol, derived from Indian lettuce, were assessed in a melanoma-bearing mice model. Mice received a single subcutaneous injection of lupeol or olive oil (solvent control) into the dorsal skin or tumor tissue. Rate of tumour growth in the lupeol-injected group were considerably lower than those in the non-treated (NT) and solvent control groups after 7 days after injection. Lupeol markedly reduced the regions strongly stained for PCNA and Ki-67 in tumor tissues relative to NT and solvent control groups. These data indicated that systemic and local administrations of lupeol inhibited tumor proliferation and induced cell cycle arrest in melanoma-bearing murine model. The data showed that lupeol might be an innovative therapeutic alternative for melanoma patients (Nitta et al., 2013).

Lupeol has shown significant growth inhibitory potential against A427 cancer cells *via* apoptosis induction. Bax and Bcl-2 expression further confirmed this apoptotic induction in lupeol-treated lung cancer A427 cancer cells. Lupeol triggered ROS generation and MMP dysregulation by inhibiting mTOR/PI3K/AKT signalling pathways (He et al., 2018). A series of new lupeol-3-urea/thiourea compounds were assessed against A549, HepG2, and MCF-7 cancer cells. All compounds exhibited superior anticancer activity compared to the parent Lupeol. Lup-20 (29)-en-3β-yl-piperazinecarboxylate-(2,4-fluorophenyl)urea (IC_50_ = 3.22 μM) exhibited the most potent anticancer activity against A549 cells, being 10 times more effective than Lupeol. Consequently, lup-20 (29)-en-3β-yl-piperazinecarboxylate-(2,4-fluorophenyl)urea served as a novel lead molecule for the advancement of additional efficacious anticancer agents targeting lung cancer (Xu et al., 2024). A separate study investigated the anticancer efficacy of lupeol on A549 and glioma C6 cells. Lupeol significantly diminished cell viability in both cancer cell lines (A549 and C6), indicating its selective anticancer action against these cell lines (Ertorun et al., 2024). The ERK pathway is a critical mechanism in the spread of lung cancer. Targeting this route is crucial in lung cancer research. A separate study revealed, for the first time, strong and specific anti-metastatic effects of lupeol on A549 cells through disruptions in the ERK signaling pathway. Lupeol effectively targeted ERK and MEK proteins, impeded cell migration, and reduced pErk1/2 and epithelial-mesenchymal transition gene expression. Consequently, it may be a prospective inhibitor of the ERK pathway in lung cancer treatment (Bhatt et al., 2021).

Androgen deprivation therapy is frequently employed in prostate cancer management (Schröder et al., 2012). Enzalutamide is a novel androgen receptor inhibitor used for castration-resistant prostate cancer treatment. Toxicity generated by enzalutamide and the potential mitigation of this toxicity was assessed using lupeol. Numerous *in vitro* and *in vivo* experimentations demonstrated that lupeol and enzalutamide engage with DNA *via* electrostatic interactions. Enzalutamide (5–20 μM) induced cytotoxicity in both cancerous (LNCaP and 22Rv1) and standard (PNT2) cells. Nonetheless, Lupeol (10–50 μM) selectively eliminated only cancerous cells. Lupeol mitigated enzalutamide-induced cytotoxicity and genotoxicity in normal cells while significantly inducing cytotoxicity in transformed cells in a dose dependent manner. Lupeol (40 mg/kg) facilitated mitigating oxidative and DNA damage from enzalutamide (10 mg/kg). Lupeol mitigated the hepatic and renal injuries generated by enzalutamide. Consequently, lupeol may serve as an adjuvant to minimize the adverse effects and augment the efficacy of Enzalutamide (Schmidt et al., 2021). In human retinoblastoma (WERI-Rb-1 and Y-79) cells, lupeol diminished cell viability and triggered apoptosis, evidenced by elevated Bax levels and reduced Bcl-2, Ki67, and survivin levels. Moreover, lupeol inhibited spheroid formation and stem-like characteristics of retinoblastoma cells. Furthermore, the ratio of LC3II/LC3I and the levels of Beclin1 &ATG7 were elevated following lupeol administration, suggesting that lupeol may stimulate autophagy in retinoblastoma cells. Inhibitory potential of lupeol on PI3K/PKB/mTOR pathway was subsequently found. Lupeol inhibited tumor growth in tumor-bearing mice, potentially due to its involvement in cell apoptosis, autophagy, and stem-like characteristics (Khan et al., 2023).

## 4 Reduced chemotherapeutic induced drug resistance

Lupeol has demonstrated latent benefits in cancer therapeutics and other human malignancies, including cardiovascular illnesses, diabetes, dermatological conditions, obesity, neurological disorders, and renal diseases. Pharmacological benefits of lupeol principally depend on its ability to enhance cellular anti-apoptotic, antioxidant, and anti-inflammatory pathways. The network pharmacological methods have helped in elucidating the pharmacological actions of lupeol and its derivatives. Notwithstanding considerable advancements in molecular pharmacology, therapeutic utilization of lupeol is constrained by its low bioavailability and inadequate understanding of its mechanism of action. Nanotechnology-assisted targeted administration and structural alterations of lupeol enhanced its bioactivity and bioavailability (Sohag et al., 2022).

Vasculogenic mimicry (endothelial-independent tumor microcirculation) has been identified in several malignancies and is believed to be facilitated by cancer stem-like cells (Pezzella and Ribatti, 2022). Dacarbazine resistance is a prevalent characteristic of melanoma, and current research indicates that this resistance mechanism is intimately associated with the development of vasculogenic mimicry (Pezzella and Ribatti, 2022; Wang et al., 2024). In B16-F10 cells, lupeol treatment exhibited sufficient cytotoxicity accompanied by a reduction in CD 133 expression. In solid tumor model, lupeol suppressed vasculogenic mimicry and angiogenesis by modifying both endothelial progenitor and cancer stem cell populations. Lupeol delayed development of rudimentary tumor microvessels by impeding maturation of bone marrow-derived endothelial progenitors. Dacarbazine therapy shown a lack of responsiveness in B16-F10 cells (*in vivo* and *in vitro* models), characterized with elevated CD 133 expression and enhanced production of vasculogenic mimicking tubes. Collectively, these findings suggested that Lupeol might serve as an effective medication in melanoma treatment by decreasing vasculogenic mimicry and potentially mitigating Dacarbazine-induced drug resistance (Bhattacharyya et al., 2019).

Cancer chemotherapeutic agents (such as sorafenib) have demonstrated diminished overall efficacy owing to several adverse effects, resulting in its frequent cessation of usage (Iyer et al., 2010). Lupeol has recently been regarded as significant potential therapeutic agent due to its reduced toxicity and improved biological activity. The Sorafenib-treated cohort exhibited a substantial elevation ROS and NOS levels, liver and renal function marker enzymes, reduced antioxidant enzymes, serum cytokines, and macromolecular damage. Furthermore, sorafenib induced oxidative stress resulted in significant cytoarchitectural damage in liver and kidneys, along with elevated expression of p53 and BAX. Notably, Lupeol combined with Sorafenib decreased ROS/RNS-induced macromolecular damage, mitigating hepato-renal toxicities. This study demonstrated the potential protective impact of lupeol against the unfavorable effects generated by sorafenib, which disrupts apoptosis and redox homeostasis resulting in tissue damage (Fatma et al., 2023).

A separate study investigated the efficacy of lupeol in augmenting the chemosensitivity of enzalutamide resistant prostate cancer cells, utilizing both *in vitro* and murine models. Combination of lupeol and enzalutamide markedly reduced viability and migration of cancer stem cells and chemoresistant cells (PTEN-CaP8 and PC3), enhanced cell cycle arrest, suppressed mRNA levels of TCF, AR, c-FLIP, and c-MYC, and inhibited tumor growth in murine model. It further mitigated enzalutamide-induced deleterious effects in prostate glands and gastrointestinal tissues. Lupeol increased the pharmacological efficacies of enzalutamide and mitigated the undesirable effects by diminishing testosterone and methionine metabolite levels. Consequently, Lupeol may serve as a promising adjuvant to enhance the effectiveness of enzalutamide-based therapies, necessitating additional investigation (Khan et al., 2023). Enzalutamide is a second-generation potent antagonist of the androgen receptor utilized for metastatic and non-metastatic prostate cancer (Rajaram et al., 2020). The emergence of chemoresistance in cancer cells diminishes the efficacy of enzalutamide (Wang et al., 2021). *In silico* and *in vitro* investigations revealed that lupeol markedly suppressed chemoresistant Du145 cell proliferation and cancer stem cell proliferation independently and in conjunction with enzalutamide. Docking and simulation analyses revealed substantial modifications in the structures of several essential proteins, including c-myc, c-flip, and β-catenin. They also markedly suppressed the transcriptional activity of all these genes. Lupeol enhanced the chemosensitivity of enzalutamide-resistant prostate cancerous cells (Maurya et al., 2022).

## 5 Enhanced efficacy using nano based derivatives of lupeol

Notwithstanding the anticancer properties of lupeol, its poor water solubility poses challenges for therapeutic application. Polycaprolactone/gelatin (PCL-GEL) nanofiber scaffold resolved the poor water solubility of lupeol. Impact of PCL-GEL-lupeol nanofibers was evaluated on cancer cell lines. To attain optimum nanofibers, a PCL-GEL solution was formulated at varying ratios (8 wt% and 4 wt%). The medication release profile verified a steady release of approximately 80% attained within 40 h. The IC_50_ value of lupeol for ACHN cell was 52.57 μg/mL and HSC-3 cell was 66.10 μg/mL. These results facilitated comprehension of creating a scaffold with an optimal dose of bioactive lupeol (6 wt%), featuring a bead-free uniform diameter that effectively binds the drug. The improved cytotoxic action through efficient diffusion and elution to the target demonstrated in this work contributed to developing a nanofiber in ongoing fight against cancer (Ravichandran and Radhakrishnan, 2022).

Lupeol liposomes, altered with Gal-PEG-DSPE, were synthesized using thin-film dispersion technique. Both lupeol liposomes and Gal-lupeol liposomes demonstrated an average particle size (around 100 nm). The encapsulation effectiveness of lupeol liposome and Gal-lupeol liposome, preserved with 15% sucrose as glycoprotein for 6 months, exceeded 80%; despite increasing particle size. In Gal-lupeol-liposomes treated HepG2 cells, cellular uptake and absorption was highest and greater apoptotic efficacy than lupeol liposomes and reduced expressions of AKT/mTOR-related proteins (p-AKT308 and p-AKT473) in comparison to free lupeol groups. Gal-NR-L had hepatic targeting effects in FVB mice. Following the treatment of Gal-lupeol-L, the mice’s liver index and liver weight were lower than those in the non-targeted group. The histological analysis revealed a more distinct lobular architecture in the hepatic tissue of mice, with more pronounced vacuoles and an increased abundance of cytoplasm following the injection of Gal-lupeol-L. mRNA expression levels of AFP, GPC3, and EpCAM were dramatically reduced in comparison to non-targeted lupeol-liposomes (Zhang et al., 2021). *Clerodendrum glabrum* is a native medicinal plant used to treat respiratory ailments, including cough, sore throat, and cold (Ogunlakin et al., 2023). *Clerodendrum glabrum* (stem and bark) contains four triterpenoids: β-amyrin palmitate, 3β-lup-20 (29)-en-3-ol, 3β-hydroxy-5-glutinene, Lupeol-3-palmitate, and stigmasterol (Cock and Van Vuuren, 2020). Remarkably, none of the compounds exhibited toxicity towards hormone receptor-positive breast cancerous cells while demonstrating differing levels of toxicity against triple-negative breast cancer and non-cancerous breast epithelial cells. Specifically, lupeol-3-palmitate (47.6 μM) and glutinol (26.9 μM) had highest inhibitory efficacy against HCC70 cells with demonstrating specific cytotoxicity towards HCC70 and not affecting the non-cancerous MCF-12A cells (Teclegeorgish et al., 2020).

Utilization of alternative chemicals, especially those administered *via* nanocarriers, is a crucial strategy for the effective therapy of cancer cells. Nanoniosomes were produced *via* the thin-layer hydration technique (Zarenezhad et al., 2024). Niosomes containing isomeldenin and lupeol triggered apoptosis *via* modifying expression levels of apoptotic genes in 3SKBr, SK-OV-3, and MCF-7 cancer cells. Combination of lupeol and isomeldenin with gemcitabine dramatically enhanced mortality of ovarian and breast carcinoma cells in niosomal forms. Consequently, it can be inferred that the nano-formulation of botanical drugs holds promise for cancer therapy (Ali et al., 2024).

Lupeol (10–25 μM) has exhibited notable alterations in cellular morphology and reduced viable cell count in human osteosarcoma cells (U-2 OS). Lupeol (5–15 μM) inhibited cell motility and invasion by reducing the expressions of β-catenin, p-p38, MMP-2/9, Ras, PI3K, p-Raf-1, N-cadherin, and pAKT in U-2 OS cells, while enhancing the expression levels of GSK3β and VE-cadherin in U-2 OS cells. Lupeol inhibited invasion through the p38/MAPK/PI3K/Akt signaling pathways in U-2 OS cells. Consequently, lupeol may be utilized in the future for the anti-metastatic treatment of human osteosarcoma cells (Hsu et al., 2019). To augment the anticancer activity of lupeol, eight derivatives of lupeol-3-succinate and seven derivatives of lupeol-3-maleate were developed. These novel compounds were assessed for their cytotoxic effects on A549 cells, HepG2 cells, and MCF-7 cells. All drugs demonstrated higher cytotoxic efficacies compared to lupeol alone. Lup-20 (29)-en-3β-yl 4-oxo-4-(pyrrolidin-1-yl) butanoate exhibited significant cytotoxicity and apoptosis against A549 cells, HepG2 cells, and MCF-7 cells. It possesses the potential to evolve into a unique anti-tumor agent by additional structural alteration (Chen et al., 2024).

In diabetes and cancer, lupeol@chitosan (LUP@CS) nanoparticles encapsulated in cellulose acetate (CA) membranes (LUP@CS/CA) were synthesized. They exhibited significant anticancer efficacy against A431 human skin cancer cells, positioning the membrane as a budding innovative therapeutic mediator for skin cancer management. The synthesized membrane displayed substantial antidiabetic prospective *via* inhibiting carbohydrate-digesting enzymes (IC_50_ = 54.56 mg/mL). These findings indicated its strong potential against skin cancer treatment and diabetes management, with considerable implications for nanobiological applications (Chen and Zhang, 2021).

Additionally, in hepatocellular carcinoma, lupeol-loaded nanoparticles increased radiosensitivity by blocking hyperactivation of RAF/MAPK and PI3K/mTOR pathways. Radioresistance constrained the efficacy of radiation for hepatocellular carcinoma. The Raf and PI3K signaling pathways facilitated the development of radioresistance in HCC. Lupeol exhibited anticancer properties despite its limited water solubility and harmful effects on normal tissues. Lupeol nanoparticles (lupeol-NPs) utilizing a PEG-PLGA diblock copolymer vector were developed, and it indicated that Lupeol-NPs counteracted the radioresistance of hepatocellular carcinoma by suppressing cellular proliferation and cell-cycle progression while enhancing cellular apoptosis through the inhibition of Raf/MAPK and PI3K/AKT signaling pathways in radioresistant Huh-7R cells. *In vivo*, Lupeol-NPs, in conjunction with radiotherapy, suppressed the proliferation of radioresistant hepatocellular carcinoma in xenogeneic nude mouse model. The Ki-67 proliferation index considerably decreased, indicating that Lupeol-NPs enhanced the sensitivity of radioresistant hepatocellular carcinoma to radiation by blocking Raf and PI3K signaling pathways (Xie et al., 2021).

**FIGURE 2:**
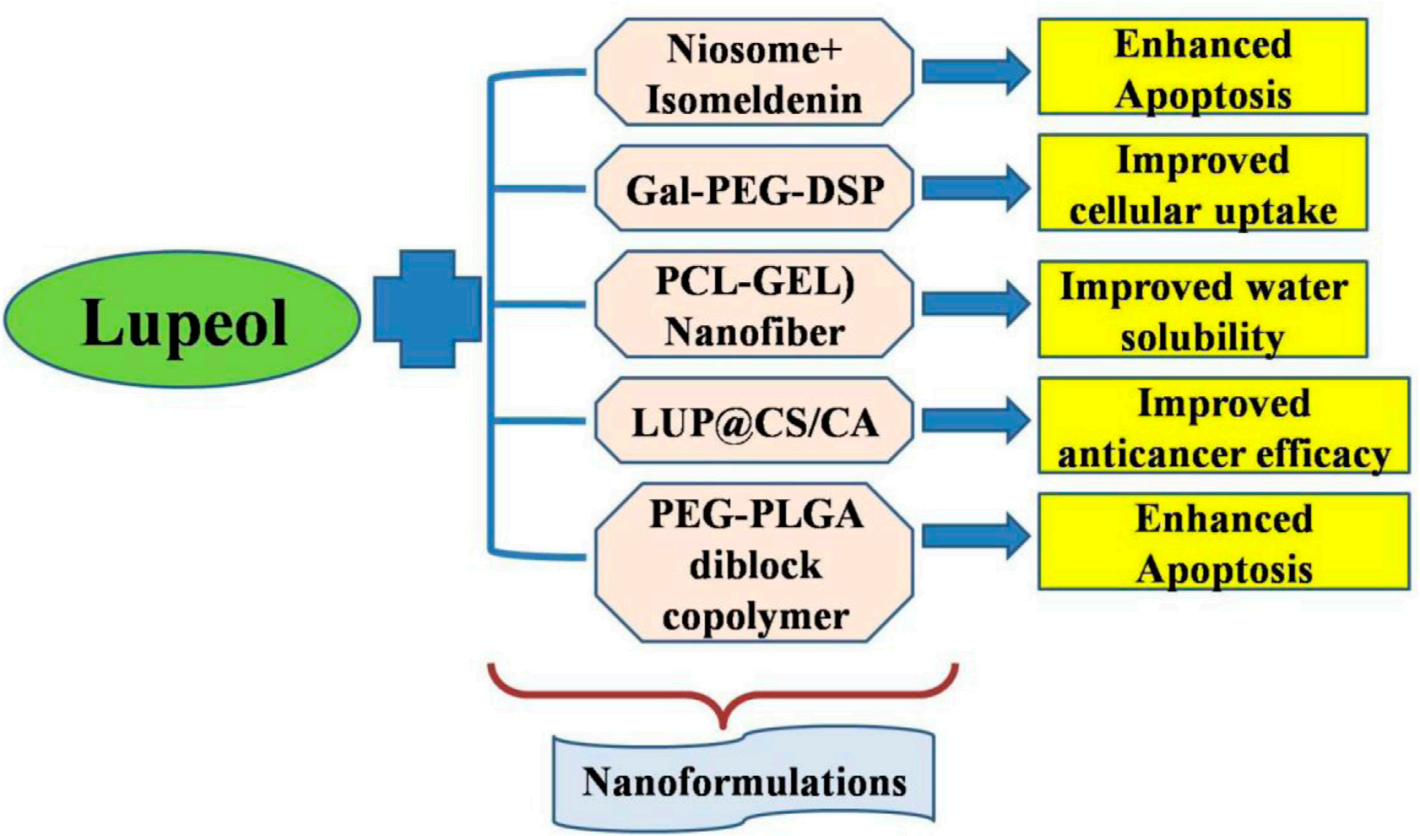
Nanoformulations employed to augment the targeted delivery of lupeol to increase its anticancer efficacy.

Collectively, our research has established a robust foundation for our comprehension of employing nanotechnology to augment the targeted and specific delivery of lupeol, aiming to position it as a formidable lead candidate for treating human carcinomas (Figure 2). Due to the limited research on the nanoformulations of lupeol, significant potential exists for developing effective nanoformulations of lupeol to improve its efficacy against human carcinomas.

## 6 Conclusions, limitations and future perspectives

Lupeol is a multifaceted pharmacological agent that protects various clinical conditions, including oxidative stress, inflammation, apoptosis, and cholesterol dysregulation. Recent research has demonstrated that lupeol can induce anticancer effects *via* many routes. Furthermore, it can demonstrate anticancer activity by suppressing cytokine release and modulating dysregulated cell signaling pathways, augmenting reactive oxygen species formation, and controlling redox equilibrium. Moreover, substantial advancements have been achieved in the nanotechnology-assisted targeted delivery of lupeol and its derivatives. Lupeol can engage with many signaling molecules and pathways concurrently, potentially resulting in off-target effects with associated hazards. Clinical experiments utilizing lupeol for the treatment of numerous disorders have thus far been unsuccessful due to its inadequate bioavailability. To this day, there have been very few or no reviews that have summarized the anticancer efficacy and its detailed mechanism of action in a variety of human carcinomas. As a result, it is anticipated that this review will contribute to the therapeutic use of lupeol to cancer treatments. Researchers are persistently striving to augment the bioavailability and bioactivity of lupeol by altering its chemical structure.

When assessing the findings, it is important to take into account the study’s numerous limitations. First of all, the use of many animal models in these type of research introduces significant variances, including changes in sample sizes, medication dosages, and rat species. These differences may lead to experiment heterogeneity and undermine the validity of the study’s findings. However, lupeol’s pharmacokinetics, distinct molecular structural properties, binding energy level, effective dose concentration, and lack of interest and accessibility have all been lacking, leading to few or unsuccessful clinical trials. Although lupeol exhibits notable pharmacological benefits, its safety remains uncertain due to the absence of toxicity evaluations in animal models. Thus, it is mandatory to assess the specific limitations in the animal models in comparison to other *in vitro* models. Given the potential and obstacles associated with establishing a lupeol-based medication, a comprehensive study is necessary in the future to investigate the efficacy of lupeol and its derivatives in cancer management and to evaluate their *in vivo* safety.
